# Nursing Section

**Published:** 1904-02-06

**Authors:** 


### The Hospital.

### Nursing Section.

Contributions for this Section of "The Hospital" should be addressed to the Editor, "The Hospital" Nursing Section, 28 & 29 Southampton Street, Strand, London, W.C

No. 906.—Vol. XXXV. *SATURDAY, FEBRUARY* 6, 1904.

### Notes on News from the Nursing World.

### QUEEN ALEXANDRA'S MILITARY NURSING SERVICE.

We are officially informed that Miss A. L. Cox has been appointed matron of Queen Alexandra's Imperial Military Nursing Service; that Sisters E. Beck, R. Osborne, and A. B. Wohlmann, with Staff Nurses S. B. Lanyon, C. G. Stronach, and E. M. Denne, have been confirmed in their appointments, and that Sisters F. M. Hall, H. T. Young, and Staff Nurse M. M. Rees have resigned their appointments.

### THE ANGLO-AMERICAN NURSING HOME AT ROME.

An account of the proceedings at the annual meeting of the general committee of the Anglo-American Nursing Home at Rome appears in another page. It will not tend to strengthen the belief that the affairs of the Home are skilfully administered. The refusal of the managing committee to consent to an inquiry into its management, on which Lady Frederick Bruce comments in a letter addressed to us, indicates a distaste for investigation which is surprising! We notice that in the annual report the managing committee say that, "viewed from a purely business standpoint, the result of the working for the year 1903 appears less satisfactory than that for 1902, there being a deficit, after deducting from the expenditure the amount received from nurses' earnings and fees from patients in the Home, of lire 2,709.35 as against one of lire 102.24." This difference, of over £100, is stated to be chiefly due to the increase in the staff, which means "increased expenditure out of proportion to increased earning power." But the managing Committee congratulate themselves that as the "Home is not run for profit, and as one of the chief objects of the Association is to supply trained nurses when called upon, they feel that they are carrying out the wishes of the founders and supporters of the Home in making sure that there will be trained nurses always available, even at some additional expense." But the earliest report issued in June 1900 refers to "the need of a nursing home for English-speaking visitors or residents," and not to the supply of nurses, as the reason why the Association was started. Again, we note that in the report for 1901 a hope was expressed that the Home would become self-supporting; and in 1902 the regular working expenses were practically covered by the fees. But in 1903 large subscriptions were required in order to cover the deficiency of over £100. It cannot, therefore, be denied that, financially, the Home is on the down grade. The encouraging sign is the letter which we gladly print from Miss Synge, a former matron of a London hospital, whose brother underwent an operation at the Home in December. His experience justifies the conclusion that the criticisms of correspondents of The Hospital have at any rate had a wholesome effect inside the Home.

### PARLIAMENT AND THE REGISTRATION OF NURSES.

Dr. Farquharson has undertaken the charge in Parliament of one of the Bills for the State Registration of Nurses. We are glad to see that an important hostile movement was initiated at a meeting of the Central Hospital Council for London, held last week at St. Thomas's Hospital, at which representatives of St. Bartholomew's, St. Thomas's, Guy's, King's College, London, Middlesex, Royal Free, St. George's, St. Mary's, University College, and Westminster Hospitals were present. It was decided by the Council to oppose any Bill in Parliament having the registration of nurses by the State for its object.

### IRISH NURSES AND REGISTRATION.

At a meeting of the Irish Nurses' Association last week, the drafts of the Bills for the State Registration of Nurses were read, and Miss Kelly strongly advocated the necessity of nurses studying the Bills for themselves in order that they might understand what the effects would be. Subsequently, Miss MacDonnell, Secretary to the Irish Nurses' Association, proposed, and Mrs. Tracey seconded, a resolution in favour of the formation of a working committee to deal with the business in connection with the Bills. It was also decided to form sub-committees throughout the country with the view of interesting the public, doctors, and nurses in the subject of State Registration.

### NURSES' CONVERSAZIONE AT ST. THOMAS'S HOSPITAL.

An enjoyable evening was spent on Friday last week at St. Thomas's Hospital, on the occasion of the Nurses' Conversazione, by a large concourse of guests who thronged the gaily-draped corridors and halls—finding their way by degrees to the central hall. Here Herr Wurm's White Viennese Band gave three musical performances which were much appreciated; Mr. Charles Bertram delighted the assembly by his marvellous sleight of hand tricks, and a remarkably good biograph was a feature of the programme. No fewer than six palmists afforded diversion in various parts of the building. But the most popular feature was the entertainment given by the "Hospital Pierrots," who sang various glees and songs of a highly amusing character; one in particular, bringing in the names and surnames of the staff and lecturers, meeting with great applause.

### ANGLO-AMERICAN HOSPITAL, CAIRO.

THE new Anglo-American Hospital at Cairo was opened on Thursday, January 21st, by Lord and Lady Cromer. The guests waited outside until their arrival, when Lady Cromer was presented with a bouquet consisting of white hyacinths and white camellias. Lord Cromer gave a short address, and Canon Butcher and an American missionary having each offered a prayer, Lady Cromer was presented with a velvet case which contained the key of the hospital door, and was asked to open it. This she did, and turning graciously towards the guests, declared the hospital opened. An inspection of the building by the company followed.

### WORKHOUSE INFIRMARY NURSING ASSOCIATION.

WE are asked to state that at a recent meeting of the executive committee of the Workhouse Infirmary Nursing Association it was decided to delay for a short time the publication of the annual report for 1903, in view of the fact that the Local Government Board has still under consideration the report of the departmental committee on nursing, and that no decision thereon has yet been made public.

### THE MATRON'S SIGNATURE ON CERTIFICATES.

AT a meeting of the Croydon Board of Guardians on Tuesday it was reported by the Infirmary Committee that a further letter had been received from Miss Vickery, late probationer nurse, requesting the Guardians to reconsider her application with regard to the matron's signature to her training certificate. The committee directed the clerk to inform Miss Vickery in reply that they could not advise the board to reopen the question. A short discussion took place by the board on the subject. Eventually, Mr. J. W. Gardiner, alluding to the interest taken by THE HOSPITAL in this matter, said he thought that it would again come before them. He was not that day prepared to move any resolution, but he would consider if he should do so later.

### A WARNING TO NURSES AND THEIR FRIENDS.

WE print the scandalous letter by a person calling himself " Mr. F. F.", forwarded to us by the secretary of a London hospital, in our correspondence column for two reasons. It will, we think, serve to put nurses on their guard against the designs of pestilent persons, whose own depravity prompts them to suspect depravity in others where it does not exist. In the second place, it shows the importance of nurses limiting their advertisements to the professional papers, whose readers are not likely to even pretend to misunderstand their meaning. The nurse, in this instance, acted with discretion. She inserted her advertisement in THE HOSPITAL, and, of course, there were no offensive replies to it. But her friends, with the best possible motives, put the advertisement in a London daily paper, with the result indicated. It is deplorable that there should be people who search the advertising columns of our lay contemporaries for evil purposes, but so long as this is the case, nurses, whose friends are anxious to protect them from the indignity of receiving grossly insulting communications, would do well to refrain from employing such channels for making known their requirements.

### ARMY NURSES IN AMERICA.

IN her account of the year's progress in army nursing in the United States, Miss Dita H. Kinney, Superintendent of the Army Nurse Corps, avers that the most notable achievement of the period was the accomplishment of the long-desired change in the transport regulations—assigning nurses to the saloon mess after the medical officers. Miss Kinney says that some of the chiefs of the various departments to which the matter was referred, objected, but "we won." There are two things which the superintendent desires and hopes to get for her staff, namely, a modification by Congress of the present law in respect to their subsistence, so that it will be unnecessary for them to contribute from their salary to have their table what it should be, and an arrangement by which when pressure of work in the hospital prevents a nurse being given her annual leave, it may become cumulative.

### A QUESTION OF TRUTH.

AN explanation has been given of the fact that the new superintendent nurse at Lewes Workhouse Infirmary gave the same age to the Lewes Guardians in applying for the appointment as she gave two years ago when she applied for a post at St. Saviour's Infirmary, Dulwich. The superintendent nurse pleads that she was advised, "to add two years to her age when she applied for the post at Dulwich, in order to obtain the appointment," and she has offered to produce her birth certificate to the Lewes Guardians in order to prove that she did not secure her present position "under a fictitious age." The offer was not accepted, the Guardians being willing to accept her word. But we hope that her experience has convinced her that it is just as wrong to try and get a post by putting years on to age as by taking them off. There can be no valid plea for obtaining an appointment by false statements, and whoever advises that they shall be made is as bad as the offender.

### MEDALS FOR ASYLUM NURSES.

AN interesting ceremony took place the other evening at the Retreat Asylum, York, when certificates and medals for proficiency in mental nursing were awarded to several members of the staff. This is the first occasion on which medals have been given, and the recipients were four nurses, Miss Phœbe E. Witcherley, Miss Susan E. Clark, Miss Ethel Mary McKew, and Miss Ada S. Sleightholme, and four attendants, Mr. Richard Thornton, Mr. John Wilson, Mr. Thomas Henry Watson, and Mr. Ernest Coulter. The medals bore the motto "Cum Bona Voluntate Servientes"—with good will during service—and the inscription: "Presented for efficiency in mental nursing." Subsequently, five *honoris causa* medals were presented as a mark of distinction to five valued officials of the institution including Miss Thomasson, the matron, and Miss Margaret Hodgson, sister-in-charge of the West Villa. The eight nurses and attendants also received the certificates of the committee for their period of training extending over a term of four years.

### NURSES FOR EMERGENCY CASES.

EIGHTEEN months ago the Notts Nursing Association was affiliated to Queen Victoria's Jubilee

Institute, and at the annual meeting, which has just been held, very satisfactory results were reported so far as the work of the nurses is concerned. But the committee announce that an increase of £100 to the annual income is urgently needed to enable them to provide additional permanent nurses for cases of emergency as well as those extra nurses who are employed during the summer months and holidays. Lord Belper, who presided, dwelt on the importance of this part of the work. He said that there were plenty of rich who would give enormous sums to large charities, "but small charities, whose work might be equally good, found it difficult to add to their revenue at short notice, and he thought that everyone concerned would have to put their shoulder to the wheel if the necessary money was to be obtained." It is only by acting upon this principle that nursing organisations can hope to extend their sphere of usefulness, or to pay their way. The unselfish and constant co-operation of every individual who is interested in the movement is a condition of success in small charities.

### VICTORIAN TRAINED NURSES' ASSOCIATION.

The third examination held in connection with the Victorian Trained Nurses' Association by the Conjoint Board of Examiners took place on December 9th and 10th at the Melbourne Hospital, and at the sub-centres of Ballarat and Bendigo. Twenty-nine candidates presented themselves, 23 of whom passed and six failed. The practical tests included bandaging, bed-making, sterilisation of sutures, and ligatures, urine testing, and mixing lotions.

### WORKING MEN AND DISTRICT NURSING.

The working men of Bishop Auckland are to be congratulated upon the admirable manner in which they manage the financial affairs of the District Nursing Association founded under their auspices 12 months ago. The first annual report shows a balance of £28 in hand. This is in striking contrast to the deficiency of £80 in the accounts of the defunct Provident Association, and the satisfactory position is likely to be augmented, as 100 men employed at the Adelaide Colliery, but residing at South Church, are about to join. As to the work of the nurses, we learn that Miss Wood paid the extraordinary number of 6,000 visits during the year. Since November she has had a colleague, whose visits from that time to the end of December were 774. It is not surprising that a nurse who works as Miss Wood has done is appreciated by working men.

### AN UNDER-STAFFED WORKHOUSE INFIRMARY.

At a meeting of the Thetford Board of Guardians a report was presented by the medical officer to the effect that Captain Hervey, the Local Government Board inspector, had expressed the opinion that the workhouse infirmary was understaffed and that a superintendent nurse, with nurses under her, should be appointed. The medical officer signified that, as there were 60 infirm and sick in the workhouse, he agreed with the inspector's suggestion. The report appears to have created amusement. The chairman characterised the suggestion as a very big order, and amid laughter said that it was evidently a patent of the inspector's. Mr. Devine, also amid laughter, observed that he supposed they would have to build another wing to accommodate the nurses, and the farcical proposal was then made to defer the matter for two years. It, however, seems to have struck some of the guardians that they might carry their hilarity a little too far. The question was therefore finally referred to the house committee, who, it may be hoped, will treat seriously a question of importance to the sick and suffering seriously.

### MAKING A NURSE PAY FOR HER ILLNESS.

At the last meeting of the Malton Guardians it was reported that the head nurse, who was recently appointed, had broken down in health. The guardians agreed that she should have a rest for a week or two, "but that during her absence she should forego her salary." We do not think that the ratepayers will altogether appreciate this piece of economy. They will be morally to blame if in order to make her financial loss as small as possible, the head nurse resumes her duties before she is sufficiently well and as a result breaks down altogether.

### NURSES AT PLAY.

By the kindness of the Committee of the Victoria Nursing Institution, Walsall, and their friends, the nursing staff were recently entertained at supper. Not only were gifts in kind and money contributed, but the pleasure of the nurses was greatly enhanced by the presence of several members of the Committee who entered fully into the spirit of the progressive games, and were perhaps surprised to find that the nurses were as proficient at play as at work. Be that as it may, the evening was thoroughly enjoyed, and the lady superintendent on behalf of the staff tendered her sincere thanks to all those who helped in any way to make the gathering so successful.

### NEW NURSES' HOME AT SALFORD.

The nurses' home in connection with Hope Hospital, Salford, which has been erected at a cost of nearly £12,000 has been formally opened by the Mayor. The building adjoins the hospital, provides accommodation for 72 nurses, and is furnished throughout in a manner which shows that attention has been paid to every detail with the view of promoting the comfort and convenience of the staff. The need for the home was imperative, as the only provision for the nurses until its completion was a number of rooms on the top floor of the hospital. These, it is now intended by the Salford Guardians, to utilise for effecting improvements in the institution itself.

### SHORT ITEMS.

The Lord Lieutenant of Cambridgeshire has given £1,000 to be specially invested, and the dividends are, in the first place, for keeping up the repairs of the Nurses' Training Buildings at Addenbrooke's Hospital.—The president of the British Gynæcological Society (Professor John Taylor) will deliver his inaugural address on Thursday, February 11th, at 20 Hanover Square at 8 p.m. Papers will be read and specimens shown by Dr. Gelston Atkins, Dr. William Duncan, Dr. Macnaughton Jones, Dr. Snow, and Mr. Spanton.—A committee of the Kingston Nursing Association, of which the Duchess of Albany is president, have presented the Princess Alice with a handsome fan as a wedding gift.

### The Nursing Outlook.

"From magnanimity, all fear above';
From nobler recompense, above applause,
Which owes to man's short outlook all its charm."

### ON MARRIAGE.

In the nursing world marriage, instead of being the aim of the majority, is only an infrequent episode. But delightful as is this freedom from the obsession of one idea that makes so many women narrow, self-centered and vain, it must be admitted that marriage is the natural fate of all and the widest expression and development of every woman's life. Therefore it seems to us that the matron or sister who looks shocked if a nurse speaks to a man, or turns the conversation abruptly if things matrimonial are discussed, is taking a wrong course. There is no reason why nurses on the whole should not look forward to the possibility of marriage, and it is certainly well that they should not take such serious responsibilities without due consideration. And all this arises from a happy little talk with a group of nurses who banded themselves together to go to the Royal National Pension Fund office and make inquiries as to whether one of their number could withdraw her contributions in order to provide a trousseau. The one most interested was so imbued with the hospital idea that a nurse who marries is a deserter from the ranks, that she was afraid to go and acknowledge her coming defection alone: therefore a time had to be chosen when two or three comrades were free, who were so wistfully wonderful over the new light in the eyes of the engaged nurse, that they thought there must be something in marriage after all. A few kindly queries, some unstinted and unqualified congratulations, and with a sigh of relief to find the subject was not taboo or regarded with scorn, and the nurses settled down to enjoy a talk. There was considerable delight in finding that having been for seven years in the Fund the engaged nurse had the right to withdraw her savings without there being any deductions—it was pointed out to her that the trustees had deliberately taken into consideration the possibilities of marriage, and that the Fund was meant to meet the needs of all who were thrifty and had an eye to the future. Should the nurse desire she could stay on in the Fund, for there are some nurses who work after marriage and add to their happiness in so doing.

The rule must ever be that the married woman's main duties are at home, for the paramount duty of a mother is to nurse and train her own children, and see that physically, mentally, and morally they have a fair start in life. But there are cases of widowhood, and cases of married life where there are no children. In the latter event the wife is apt to find her days empty if her husband is much away, and her heart turns with longing to the thought of the children's ward. Now in this country of ours where the women outnumber the men by many thousands, and therefore marriage cannot be the lot of all, there are also many thousands of neglected children who sadly need the care of a sensible and loving woman. And if anyone can properly take charge of the most unhappy of these little ones, surely it is the nurse who has been trained in patience and discipline and sympathy, and trained also in the knowledge of hygiene and physiology? So it seems to us that there are many openings for the married nurse if she have no children of her own. She can work for the Invalid Children's Aid Society, the School Board or some cripples' home. She can take up daily work—and the visiting nurse is a great blessing to the lower middle class who cannot afford the expensive hospital nurse staying in the house; it is a movement which is bound to develop as the increased teaching and training brings increased fees and demands from the nurse-student of the future.

And so the nurse need not be a deserter, can still take her place as one of the world's workers for others, either with her own children in her own home, or through the medium of public service if she be not granted this satisfaction. There is no doubt that a nurse ought to make the very best of wives; having an interest and an income of her own she is not likely to marry except under the influence of strong affection. All the experience and training of hospital life go to develop all that is best in a good woman. Grant this, and grant that the "happy homes of England" are the main element of her power and her greatness, and it seems a pity to make matrimony an obnoxious subject to the unfortunate probationer. It is doubtless very annoying to the authorities when they have trained a good nurse and she is fulfilling excellently her position, that she should go and throw them over for some one mere man. But with due length of notice the nurse has time to reflect and the committee has time to fill her place; it is not difficult with so many women eager for work to keep the gaps filled, and the nation profits when the well-trained woman marries. To be a mother is even more important than to be a nurse. Therefore, let the subject of matrimony ever be dealt with with the honour due to its importance, and without either the feeble facetiousness of the joker or the contemptuous scorn of the crabbed old maid. Let it be acknowledged that it is a possible occurrence in our "Nursing Outlook," for, as Robert Louis Stevenson has said, "Place them in a hospital, put them in a jail in yellow overalls, do what you will, young Jessamy finds young Jenny." And for our part we say, "And a good thing too! for no good can come to fighting against the common law of life. And just as it is her very womanliness which makes a nurse a good nurse, so will it help to make her a good wife."

### Lectures on Ophthalmic Nursing.

By A. S. Cobbledick, M.D., B.S.Lond., Senior Clinical Assistant and late House-Surgeon and Registrar to the Royal Eye Hospital.

### LECTURE XXVII.—DISEASES OF THE EYELIDS.

Only the common affections of the lids will be dealt with in this lecture.

*Hordeolum* or Stye.—This is such a common affection, and its characteristics are so well known that it scarcely needs description. It begins as a small red swelling in the neighbourhood of one of the eyelashes, and is accompanied by itching and subsequently by heat and considerable pain. The swelling increases and may extend over the greater part of the lid. Eventually pus is formed, and, when it points it can be opened, and the small abscess evacuated by pressure. There is a great tendency to recurrence, no doubt due to the micrococci finding their way into surrounding hair follicles.

*Treatment* consists, in the early stage, of ice-cold applications. If this does not abort the inflammation, hot boracic fomentations should be applied frequently; they relieve the pain and hasten the abscess formation.

To prevent recurrence, a mercurial ointment should be applied to the lid night and morning for some weeks. If any error of refraction is present—as is usually the case—it should be corrected, and glasses worn. Eye-strain in some way produces a congested state of the margin of the lids which, no doubt, predisposes them to infection by cocci.

*Meibomian cysts.*—These also form swellings—sometimes red—in the lids; they are situated more deeply in the lid and usually at a greater distance from the margin of the lid than are styes.

These may be classed as non-inflammatory and inflammatory. The former make their appearance by producing a slight elevation on the surface of the lid, which is not movable and which is hard to the touch; the skin of the eyelid is freely movable over its surface. In time this gradually increases in size and produces an irregularity of the ocular surface of the lid, so causing discomfort by its constant movement over the sensitive cornea.

The causation is obscure; they occur chiefly in adults, and are somewhat prone to recur. When these cysts become inflamed the swelling increases rapidly in size, and is accompanied by much pain and discomfort. The diagnosis is seldom difficult; occasionally there may be some doubt when the swelling is great and most marked near the edge of the lid, but inspection of the ocular surface of the lid and the position in which the abscess is tending to point, usually settles the question.

*Treatment.*—In the non-inflammatory cases it is well to leave small cysts alone, as they are not easy to open with certainty and are very apt to recur in a short time. When they grow larger, they can be located by the appearance of the ocular surface of the lid, where they produce an alteration of the normal red colour of the lid, forming a small greyish or yellow circle. No drugs are of any use in curing the condition. The cysts must be opened and the contents evacuated.

Two or three installations of cocaine, 10 per cent., render the conjunctiva anæsthetic; the lid is everted and the cyst opened with a pointed knife from the ocular surface; by this means there is no disfigurement from an external wound. The incision should be made vertically, and should extend along the whole of the presenting surface. The contents may be scraped out with a curette, or expressed by the finger and thumb. The cavity which remains fills with blood clot, so that it is some days before there is any diminution in the size of the swelling: patients should be informed of this fact in order that they may not regard the operation as unsuccessful. In the non-inflammatory cases a soft, granulation-like tissue is removed from the cyst; in the inflammatory cases this granulation tissue is mixed with a yellowish muco-purulent fluid. The after treatment consists in keeping the conjunctival sac clean by using boracic acid lotion.

*Blepharitis* is a very common complaint, especially in delicate strumous children who suffer from hypermetropia and astigmatism. In slight cases there are always some scales adherent to the eyelashes, which can, however, be easily removed; all degrees of severity are met with between this and the formation of a thick crust along the margin of the lids, which on removal leaves a red, and in parts bleeding, surface, devoid of lashes. Severe cases are very troublesome to cure and recur with great persistency.

A large percentage of cases of blepharitis are associated with errors of refraction, and all cases should be carefully tested, and if necessary lenses should be ordered. The *treatment* consists in removing the scab as it forms by means of an alkaline lotion, *e.g.*, sodium bicarbonate, gr. xv. ad. ℥j., carefully drying the lid and then applying the ung. hydrargyri ox. flav. (2 to 5 per cent.); the latter should be vigorously rubbed into the margin of the lid. If this treatment is applied night and morning for a few weeks, most cases are improved if not cured. If the child is markedly strumous, the general health should be attended to, and, when possible, country life should be advised.

Entropion, ectropion, and ptosis are the three remaining most common affections of the lids.

*Entropion* consists of an inversion of the margin of the lid, so that the lashes are brought into contact with, and cause irritation of, the cornea.

This usually happens to the upper lid, and is caused by the contracting scar left after repeated attacks of granular ophthalmia; if the upper lid in such a case is everted, a white horizontal scar can always be detected. If the lashes are allowed to continually irritate the cornea, the latter becomes dull and vascular, and the vision much impaired. The treatment consists in a plastic operation so planned as to evert the margin of the lid and draw the lashes away from the eyeball.

*Ectropion* consists in undue eversion of the lids, usually the lower. A cicatrix caused by a burn or injury is not an uncommon cause. In old people it is not uncommon from atrophy of the orbicularis muscle. The chief inconvenience of this condition is the epiphora caused by the punctum being drawn away from the ocular conjunctiva. In slight cases, slitting of the duct and the use of an astringent lotion may relieve the condition. In more severe cases, a plastic operation is necessary.

*Ptosis* is the name applied to inability to raise the upper lid so that the palpebral fissure is much narrowed.

It may be congenital, and then affects both eyes. When one eye only is affected, it is probably either due to some central nerve lesion or a blow on the neck.

The first class require a plastic operation; many of the two last types recover under appropriate treatment.

### Royal British Nurses' Association.

### REGISTRATION OF NURSES AND OF PRIVATE NURSING HOMES.

A SPECIAL general meeting of the Royal British Nurses' Association was held on Tuesday to consider the draft of a bill to provide for the better training and registration of nurses, and for the Voluntary Registration of Private Nursing Homes. The draft was based upon the synopsis which was laid before the special general meeting last month. There was a large attendance of nurses and doctors. Mr. E. A. Fardon was in the chair, and with him on the platform were Dr. Comyns Berkeley, Hon. Medical Secretary, Mr. Langton, Hon. Treasurer, and Miss Hobbs, Secretary.

The main divergences from the synopsis were as follows:—

The first clause of the draft provided that after January 1st, 1908, no woman should habitually and for gain practise as a registered nurse unless she were registered under the Act, and that any woman so acting without registration would be liable on summary conviction to a fine not exceeding £10. To this clause Dr. Comyns Berkeley added an amendment which was passed. It provides that no woman shall be deemed to be practising as a nurse habitually and for gain within the meaning of the Act who in the course of the training is in *bonâ fide* employment as a nurse at any hospital, Poor-law infirmary, or other recognised institution, and that no woman who is employed to attend upon persons suffering from incurable diseases or who are old or infirm shall be deemed to be practising as a nurse. The conditions under which a woman shall be registered were unaltered, as was also the provision for existing nurses, with the exception that the year of grace was extended to two years. The regulations for nurses trained in the colonies were unchanged. The constitution of the Central Board had, however, been much modified, as it was found that its size was too great to be workable. The numbers were therefore reduced from 31 to 21, and were thus apportioned: Three registered medical practitioners to be appointed for terms of three years, two to be appointed by the Lord President of the Council and one by the British Medical Association. Three persons to be appointed for terms of three years by the Lord President of the Council, representing England, Scotland, and Ireland respectively. Three representatives to be appointed for terms of three years, one to be appointed by the Director-General of the Navy and the Medical Director-General of the Army conjointly, one by the Royal British Nurses' Association and one by the Queen Victoria Jubilee Institute for Nurses. Five fully-trained nurses who shall be matrons or lady superintendents of hospitals or Poor-law infirmaries with training schools attached, one to be elected by the matrons and lady superintendents of metropolitan hospitals, one by those of the metropolitan Poor-law infirmaries, one by those of the provincial hospitals, one by those of the hospitals in Scotland, and one by those of the hospitals in Ireland, all with training schools attached. Six fully-trained nurses to be elected for terms of three years, two to be elected by nurses on the register resident in the metropolis, one of whom shall be a mental nurse; two by nurses in the provinces and Wales and one each by nurses resident in Scotland and Ireland.

An amendment was brought forward by Dr. Biernaki for the addition of a nurse from a fever hospital, and this being passed, raised the number of fully-trained nurses to seven. Under the regulations as to retirement was a proviso that the nurse-members of the board were not to be elected until the end of six months from the first registration of nurses.

The duties of the Central Board were as set forth in the synopsis, with the exception of the deletion of the paragraph regulating the admission to the register of women already in practice, this being included under the first clause. An amendment moved by Mrs. Bedford Fenwick, and carried, provided that no rules should be framed or adopted till the Central Board was fully constituted. It was stated that the rules framed by the Central Board would only be valid if approved by the Privy Council.

A discussion took place upon the clause headed: "Appeal from decision of the Central Board," and an amendment was carried. The clause as amended provides that any woman thinking herself aggrieved by any decision of the Central Board removing her name from the register, may appeal therefrom to the High Court of Justice within six months of the notification of such decision, and that the Central Board shall not suspend nor remove any member's name from the register without sending to such member a statement in writing of the conduct imputed to her, and without affording her an opportunity of giving an explanation in writing or in person as she may elect. No alteration was made in the clause regarding fees and expenses. The next clause dealt with the Nurses' Register, and the following one regulated the badges. Then came the clauses touching upon the inspection and registration of private nursing homes, providing that upon the written request of the person owning or managing a private nursing home the Central Board shall inspect it and register it if the regulations of the board had been complied with. The Central Board is to be empowered, in respect to the registration of private nursing homes, to frame regulations, appoint visitors, fix and collect fees for registration, keep and publish annually a register of these homes, and decide upon the removal from the register of any home where the regulations were not observed, and to decide upon the restoration to the register of any home so removed. The owners or managers have also the right of appeal as set forth above.

The clause headed "Appointment of Secretary and supplemental provisions as to registration" was the same as in the synopsis, as was also the penalty for obtaining registration by false representation or for wilful falsification of the registers. It was stated that any woman deeming herself aggrieved by any determination of any court of summary jurisdiction under this act might appeal to the Court of Quarter Sessions.

A clause headed "Definitions" stated that the term "nurse" meant a man or woman registered under the Act. The Act is to be cited as the Nurses' Registration Act.

The various clauses of the draft thus amended having been adopted, Dr. Comyns Berkeley moved that the draft bill, as amended, be approved, and that the executive committee be directed to take such steps as they may think necessary to have it submitted to Parliament. This resolution was passed and the proceedings came to a conclusion.

### Death in Our Ranks.

MISS FRANCES BELL died on Tuesday, of enteric fever, after fourteen days' illness, aged 28 years. She was trained at the City Hospital, Edinburgh. Miss Bell had for the past nine months been charge nurse at the Isolation Hospital, Whitburn, near Sunderland.

### The Anglo-American Nursing Home at Rome.

### ANNUAL MEETING OF THE GENERAL COMMITTEE.

THE annual meeting of the Anglo-American Nursing Home at Rome was held on Wednesday, January 27th, at the British Consulate.

There were present the members of the managing committee, about 30 subscribers, a few of the general public, and the matron.

Mr. A. C. Trench was elected chairman in accordance with Article XX. of the Memorandum of Articles of Association of the Incorporated Anglo-American Nursing Home, which enacts that the general meeting shall elect its own chairman.

The minutes of the last general meeting of February 10th, 1903, were read and passed.

The Chairman said that, previous to the business in hand, he wished to report that on his visit to the Home a day or two ago every bed was occupied, every nurse not employed in the Home was out, and everything betokened care and skill in the direction of the Home. He then spoke of the letters which had appeared in THE HOSPITAL, saying that they had been inserted because Sir Henry Burdett wished to sell his paper: that the Embassy people had not been very nicely treated by some of the writers, and that there were some people, who, finding that they could not run the show as their own, did all they could to hamper it. He next alluded to a letter from a lady signing herself "May Dunlop," the purport of which was that the writer had been asked to give evidence against the Home, and that she had refused to do so and had sent £5 as a donation to the funds. He also referred to another letter from Miss Synge, who had just resigned a nursing or hospital appointment in London, saying how pleased she was with the accounts she received from her brother, who had undergone an operation for appendicitis in the Home, though at first she had opposed his going to the Home owing to the correspondence in THE HOSPITAL.

In conclusion he said that, living only three months of the year in Rome, he had placed his resignation in the hands of the committee, and he was quite ready to retire whenever they wished it; also that as Colonel Lamb, Mrs. Abbott and Mr. Morgan, were to retire this year he proposed them for re-election. He presumed that as all present had received and studied the report it need not be read unless the meeting desired it.

### QUESTIONS ON THE REPORT.

The report was therefore not read. Lady Frederick Bruce, Miss Bousfield, and Miss Bulwer remarked that they had several questions and comments to offer, and indicated the paragraphs in the report which they desired to speak about. In the first paragraph they noted that 36 patients had been received. It was asked what were the diseases treated? To this the reply was that no synopsis would be given by the committee, as patients objected.

Questions were asked by Lady Frederick Bruce and Miss Bousfield regarding a case of diphtheria and one of measles, both of which though excluded by the rules of the Home, were currently reported to have been received in the institution. The matron was questioned as to the diphtheria case—she "did not think so," "was not sure," and finally said "No." Asked about the case of measles the matron was understood to give a similar reply, and the Chairman remarked that this was a proof that incorrect statements were circulated.

In the report two free beds are alluded to as reserved for American and English patients. It was asked why these beds were allowed to be occupied by patients at a reduced charge? The reply was that they were empty, so the committee thought that they might earn a little.

The report stated that the adoption of a uniform had been authorised by the committee.

Lady Frederick Bruce said that the use of an outdoor uniform was a very unwise departure, as nurses so dressed were much disliked by managers of hotels, where their work chiefly lay, as it drew attention to the fact of illness in the house. The Chairman replied that the idea of hotel managers objecting to nurses in uniform was new to him.

Lady Frederick Bruce wished to know how the bad debt of nearly 1,000 lire (about £40) had been contracted? The Chairman preferred to say nothing about it, as he was anxious not to prejudice legal proceedings which were pending.

### THE DECREASE IN THE REVENUE.

Miss Bulwer said that the result of a careful comparison of the Home funds was hardly as rosy as the committee sought to make out. There was a falling-off of 292 lire (almost £12) in the total of annual subscriptions for general expenses. The decrease was still more marked in the donations of the two years, there being 28 donors in 1903 as against 139 in 1902, a decrease of 111. The amount of the decrease was 2,869 lire (about £115). The final result was a diminution in donations and annual subscriptions combined of 3,161 lire (about £126). All this pointed to decreased general interest. Fees to nurses to the amount of £20 had to her knowledge been paid to another institution, which would otherwise have been earned by the staff of the Anglo-American Nursing Home. These remarks were received in silence, the Chairman merely observing that as the Home was now on its feet they did not need to ask for donations, and he for one had ceased to solicit them as formerly.

### AN UNUSUAL GRANT.

Lady Frederick Bruce commented on the strange grant of a sum not exceeding 100 lire to defray the expenses of nurses' visits to museums and galleries, and Miss Bousfield said that she for one would not subscribe to the funds of the institution if any portion of them were to be applied in this way. The Chairman said that the matter had been discussed in committee and there had been a difference of opinion.

### THE PROPOSED ENLARGEMENT.

Lady Frederick Bruce, referring to the proposed purchase of land adjoining the present house, asked how the committee could expect countenance for incurring further expenses when they had finished the year in debt, and how cheaper accommodation could be provided in a new building than in the existing house? The Chairman replied that unless a generous donor gave the necessary sum the ground could of course not be secured; but he did not explain about the accommodation.

### THE THANKS TO THE DIRECTRESS.

Miss Bousfield, alluding to the expressions of thanks to the directress in the report, distinctly objected to associate herself in such a vote after the matron's conduct in the past to Dr. Burton Brown, and the complaints of her management, which had never been properly investigated. The Chairman said that they had, but Miss Bousfield maintained that the investigation had been refused. Finally, after some argument, it was decided to put it to the vote whether the question should be reopened, seeing that there were several present who held that inquiry had been stifled. The majority decided against re-opening the question.

ALLEGED DISCOURTESY TO DR BURTON BROWN.

Dr. R. Wilkinson remarked that he much regretted the decision, since he considered, as a medical man, that Dr. Burton Brown had been very discourteously and badly treated.

The three retiring members of the committee were then re-elected, two other names proposed being rejected. Dr. Wilkinson explained that he abstained from voting for the re-election of the former, because he thought that it would have been advisable, after all the friction which had taken place, that the whole of the managing committee should have retired and new members taken their places.

SIR RENNELL RODD AND THE FREE BEDS.

Lady Frederick Bruce said that in connection with the statement in Sir Rennell Rodd's letter to THE HOSPITAL, that no free-bed patient had ever been refused, she was puzzled, as one member of the managing committee had told her that a consumptive case had been refused, because the Italian sanitary rules precluded the reception of such a case in the Home. After some consultation, the Chairman announced that the true reason of refusal was the applicant's consumptive condition, but it had not been given in order not to hurt his feelings. Miss Vansittart's letter to THE HOSPITAL was commented upon by the Chairman, but she maintained her proposition, viz., that Sir Rennell Rodd had said that no free-bed case had ever been refused since the opening of the Home, whereas one had been refused.

MISS BULWER AND "THE HOSPITAL."

Miss Bulwer then said:—It has come to my knowledge in connection with the correspondence in THE HOSPITAL, that a member of the Managing Committee of the Home has stated that I was the originator of the controversy, and very possibly, also, the writer of some of the anonymous letters which have appeared in that paper. The only letter I have contributed to THE HOSPITAL on this subject, was that appearing over my signature on December 26th, in which I stated that I had been "an interested reader though not a writer." This statement was received in silence, and the meeting broke up after the usual complimentary votes.

### Presentations.

ANCOATS HOSPITAL, MANCHESTER.—Miss Robinson, who resigned her post of sister in Ancoats Hospital after holding that position for 11 years, was recently the recipient of a case of cutlery from the board, a writing bureau from the medical staff, a silver cake basket from the nurses, a case of silver spoons from the ladies' committee, and table linen from her matron.

VICTORIA NURSING INSTITUTION, WALSALL. — Miss Holloway, lady superintendent of Walsall Victoria Nursing Institution, has been presented by the committee and a few friends with a purse of gold, in recognition of her work and devotion to the interests of the institution during the last three years.

### Where to Go.

CONCERT, 11 Airlie Gardens, Campden Hill, Kensington, in aid of the new Hospital for Women, Euston Road, Tuesday, February 9th, at 3 P.M.

### Everybody's Opinion.

**[Correspondence on all subjects is invited, but we cannot in any way be responsible for the opinions expressed by our correspondents. No communication can be entertained if the name and address of the correspondent are not given as a guarantee of good faith, but not necessarily for publication. All correspondents should write on one side of the paper only.]**

ANGLO-AMERICAN NURSING HOME AT ROME.

MISS K. SYNGE, late matron of the Royal Hospital for Diseases of the Chest, London, writes: May I ask you to insert these few lines? In November last it was found necessary for my brother, staying in Rome, to undergo a somewhat serious operation. Having noticed the persistent campaign waged against the Home in THE HOSPITAL, I urged that he should return to London. Being too ill to travel and hearing a most satisfactory account of the Home from those on the spot competent to judge, he underwent the operation there on December 1st. May I quote from his last letter to me dated January 19th. "After a stay of several weeks in the Home, I should like to set right any wrong impressions you may have had regarding its efficiency. The nursing has been excellent, and I cannot speak too highly of the personal attention and kindness shown me by the directress. The whole atmosphere of the Home seems to betoken a thoroughly well-working establishment."

LADY FREDERICK BRUCE writes from Rome: As one of the dissentient minority at the annual general meeting of the Anglo-American Nursing Home, may I be allowed space in your valuable paper to ask a question which the chairman of the meeting brushed aside as "hypothetical," and also to state my reason for requesting that an inquiry should be instituted into sundry complaints that have been made as to the management of the Home. My question was as follows:—"Is any doctor justified in sending patients to a home in which he has good reason to believe his instructions have not been carried out in past cases?" My reason for asking for an inquiry is that, in spite of the declaration of the committee that the subject has been threshed out, no real inquiry has ever been made into the complaints of the entire nursing staff at the close of the season 1901–1902. The efforts of those whose sole object was to get at the truth of the allegations were frustrated by an obedient majority at the special meeting held on December 10th, 1903, no record of that meeting having ever come to light, and no reference having been made to it in the printed report of the Home for 1903. That same obedient majority upheld the chairman on January 27th, 1904, when he refused to listen to my request that a thorough inquiry should be made. Naturally it is much easier to leave matters in *statu quo* than to face complaints the full inquiry into which would involve great exertion in the way of reform, and possibly some unpleasantness.

IN THE INTEREST OF NURSES.

THE SECRETARY of a well-known London hospital writes: A former nurse of ours inserted an advertisement in your journal, and friends who were interested in her welfare inserted another in a daily London paper.

> TRAINED NURSE desires daily CASES. Medical or surgical. In or near London. Highest testimonials. NURSE, 25 Mundania Road, Honor Oak, S.E. (375)

The following is one of the replies to the advertisement in the daily paper:—

> Mr. F. F. having seen Nurse's advertisement in to day's *Daily Graphic* concludes that it is meant for a covered-up massage advertisement, and if so, he will be very pleased to hear fully from her as to terms both for a patient to call upon her or for her to come to the patient. F. F. would also like to know if she has a young hospital nurse to assist her in rubbing the poor fellow. Photos of self or assistant if sent will be most faithfully returned within three days, and letters will of course be treated as strictly private. F. F. has lost a most charming nurse who used to attend to him thoroughly, she having got a good housekeeper's appointment in Africa to a wealthy old boy. Kindly address F. F., c/o Messrs. George, Stationer, Swallow Street, Piccadilly, W. January 19th, 1904.

Of course I know that nothing can be done to stop this form of correspondence. The real meaning is obvious. I cannot refrain from letting you see the letter, as you have at all times been keenly alive to the interests of nurses.

### THE QUESTION OF PRESENTS.

"A Nurse" writes: In reference to the letter from a nurse on the question of presents, to which Sir William Bennett referred in his address on Private Nursing in The Hospital of January 23rd, may I say that it must be a matter not merely of regret but of humiliation to most of the nursing profession that such a letter should ever have been written. To the writer I commend a quotation from that well known book "Dr. Anderson's Medical Nursing," in which he says that no good nurse will ever expect the slightest recognition of her work from her patient. After many years of hospital life we lose sight of that idyllic thought that sick nursing is nothing but smoothing pillows, but the spirit of our work that we go to relieve suffering still remains, and if we can do that our work brings its own reward. Of course I do not deny that it is a real gratification to us if our patients appreciate us, but that is very different to expecting a present or a legacy; and I endorse the opinion of the entire institute to which I belong when I say that the letter which Sir William Bennett quoted was a disgrace to the writer.

### ASSISTANT NURSES IN WORKHOUSE INFIRMARIES.

"A Poor-Law Matron" writes: May I be allowed again to say in reply to Miss Lee's inquiry, first if there were not the supply there would not be the demand for assistant nurses. It would mean that boards would have to pay higher salaries, I grant, but I can hardly think because they could not get assistant nurses at £20 or £25 they would revert to old customs or do with less attendance on the sick. Then, surely, if a nurse of three years' training is a nurse in the true sense of the word, she would work under a fellow nurse placed in superiority over her, for at least a time, wherever the sick wards might be, till she felt she herself was experienced enough for a higher position, and anyway, if out of work she would be fully fitted for the world and up to date. What I look at as most serious is the position of the assistant nurse, launched into the nursing world not fully equipped, simply for want of her three years' certificate; often quite as good a nurse as a fully-trained, but debarred from taking higher positions, all her life. I know a few exceptional lucky ones do get on, but they greatly miss the secure feeling a certificate would give them.

### SIR WILLIAM BENNETT AND PRIVATE NURSING.

"A Frequent Contributor" writes: I think that many nurses will agree in protesting against Sir William Bennett's dictum, that "decency" and "indecency" are words which should be expunged from a nurse's dictionary. It is rather a difficult subject to discuss, but it seems quite simple. The chief objection urged by medical men against the appointment of women to house surgeons' posts at general hospitals is that there are cases which it is not suitable for a woman to treat. Surely the same argument applies equally to the nursing of these patients? Again, in hospitals there are cases termed "screen cases" and dressings recognised as "doctors' dressings," when, if the patient is conscious, a nurse's presence is neither desired nor admitted. Why should such cases be deemed unsuitable for a woman to attend to in hospital and yet fit for a private nurse? I have nearly always found doctors, especially the senior men, most particular in dispensing with a nurse's services when attending them, and have always felt that they would never ask such service except when absolutely necessary. If a man of Sir William Bennett's standing publicly declares that "every case of disease or sickness is a case with which a nurse must be prepared to deal," what protection can she hope for against the lazy house surgeon, fortunately not often met with, who wants to make the nurses do all the dressings he alone should touch, to save himself trouble? I know from personal experience, having once worked in a ward with "old-fashioned nurses" with a contempt for modesty, that the house surgeon, though he may avail himself willingly of a nurse's readiness to dress his cases, does not have much respect for a woman who "does not care what she does for a patient," and I am quite sure no such woman can have any respect for herself. I may say that I have been nursing for ten years, the last seven as sister of male wards in civil and military hospitals and I think a nurse should always respect both her own and her patients' modesty and should never lower her womanhood and her profession by outraging it. No one can divest herself of her natural instinct of decency and retain her refinement and self-respect. I am quite willing, however, to admit that "necessity knows no laws" and that circumstances may arise where nothing but the patient's needs should receive consideration. But in a hospital where male dressers and doctors are working and in a civilised country where male nurses are available, women should neither be asked nor permitted to nurse the cases alluded to.

### Appointments.

[No charge is made for announcements under this head, and we are always glad to receive, and publish, appointments. The information to insure accuracy should be sent from the nurses themselves, and we cannot undertake to correct official announcements which may happen to be inaccurate. It is essential that in all cases the school of training should be given.]

Bucklow Union, Manchester.—Miss Agnes Dudley has been appointed superintendent nurse. She was trained for three years at Shoreditch Infirmary, where she has since been ward sister. She holds the L.O.S. certificate.

Fulham Infirmary.—Miss Laura Mary Cutler and Miss Leonora Kendrew have been appointed sisters. Miss Cutler was trained at St. Marylebone Infirmary, where she was afterwards staff nurse, ward sister, and theatre sister; she has also been charge nurse at one of the fever hospitals under the Metropolitan Asylums Board. Miss Kendrew was trained at St. Marylebone Infirmary, where she was afterwards staff nurse, ward sister, and theatre sister; she has also been charge nurse at the South-Eastern Fever Hospital.

Kingston-upon-Hull Sanatorium.—Miss Sophia Norris has been appointed night superintendent. She was trained at Queen's Hospital, Birmingham. She has since been nurse at the City Fever Hospital, Birmingham, private nurse in London, and sister-in-charge of the female surgical ward at Queen's Hospital, Birmingham.

Scarborough Cottage Hospital and Convalescent Home.—Miss Champley has been appointed matron. She was trained at the Royal Infirmary, Edinburgh, and was afterwards ward sister and home sister at the Hull Royal Infirmary and hospital matron of the Winburg Concentration Camp, South Africa.

Stirling District Asylum, Larbert.—Miss Catherine Borthwick and Miss Jean McGrigor have been appointed assistant matrons. Miss Borthwick was trained in general nursing at the Royal Infirmary, Edinburgh. Miss McGrigor was trained at the Victoria Infirmary, Glasgow, and has since been charge nurse in a private nursing home, and sister of the surgical wards at the Royal Alexandra Infirmary, Paisley.

West Cornwall Miners' and Women's Hospital, Redruth.—Miss Ellen Munday has been appointed charge nurse of the medical and surgical wards. She was trained at St. Mary's Hospital, Manchester, and has since been nurse at St. Paul's Cray, Kent, and at Liskeard Cottage Hospital.

Westminster Cottage Hospital, Shaftesbury.—Miss Winifred Jones has been appointed nurse matron. She was trained at the Taunton and Somerset Hospital, and has since been staff nurse at Newmarket Accident Hospital, matron of Sidmouth Cottage Hospital, and matron of Abergavenny Hospital. She holds the certificate of Apothecaries' Hall for dispensing.

### Echoes from the Outside World.

### Opening of Parliament by the King in Person.

ON Monday the King and Queen left Windsor and returned to London in order to be present at the State opening of Parliament. The weather on Tuesday was not auspicious, but in spite of the rain, large crowds assembled at points along the route to witness the Royal procession. The King and Queen left Buckingham Palace at 1.28, and had an enthusiastic reception all along the route, reaching the House of Lords at 1.50. A great number of people assembled in the Royal Gallery at Westminster, through which their Majesties passed to the Upper Chamber, and the scene at this stage was magnificent. The King read the speech from the Throne, which was of considerable length, the main features being our relations with foreign Powers, and the legislative programme for the Session. Bills for dealing with the evils of immigration of criminals and destitute aliens, licenses for the sale of intoxicating liquors, the hours of employment in shops, and other matters were foreshadowed. Their Majesties left the House of Lords at 2.20. The Prince and Princess of Wales were among those who participated in the ceremony.

### The Royal Wedding.

THE wedding-dress being prepared for Princess Alice of Albany, who is to be married next Wednesday, is specially beautiful. It is of a new material known as satin *charmeuse*, uniting the softness of crêpe de chine with the richness of satin. The skirt opens in front over a narrow petticoat, trimmed with small garlands of embroidery and fine chenille fringe. The train lies a yard and a half upon the ground, and is embroidered down the sides and all round in finest chenille with raised roses of white chiffon. The bows which catch the skirt in places are of white velvet ribbon studded with brilliants. The bodice is trimmed with an embroidered fichu and the sleeves are of white chiffon. A long trail of orange blossom and clematis hangs down one side of the skirt and a small cluster will be worn in the hair, with a splendid diamond tiara-ornament in the form of wheat-ears. The veil to be used is of finest Honiton lace, and was worn by the late Duchess of Teck and the Princess of Wales at their weddings. The bridesmaids will wear pale blue crêpe de chine with insertions and flouncings of lace. A broad folded sash of blue crêpe will be folded round the waist. All the bridesmaids will wear wreaths of forget-me-nots in their hair, with a single spray of white heather.

### War Office Reform.

THE King has approved of the issue of the report of the War Office Reconstruction Committee, and in accordance with the recommendation of the committee the Government, with his Majesty's approval, have decided to appoint an Army Council framed on the model of the Board of Admiralty. The council will be constituted by Patent; the office of Commander-in-Chief will cease to exist; and a new post will be created, viz. that of Inspector-General, whose principal duty will be to inspect and report on the efficiency of the military forces under the control of the Home Government.

### Safety of the East African Syndicate Party.

IT is officially announced that the Foreign Office has received from Sir Charles Eliot, his Majesty's Commissioner in the East Africa Protectorate, a telegram as follows:—"News received from East African Syndicate Party, dated January 20th, reporting all well, and expected to reach Munias middle of next month." This party was reported to have been cut up in the neighbourhood of Lake Rudolph, and the intelligence caused many inquiries at the Foreign Office, and at the London offices of the East African Syndicate. All anxiety in the matter has now been effectually and happily dispelled.

### Poisoned by French Beans.

A SAD fatality has happened at a ladies' cookery class at Darmstadt. The lady superintendent opened a tin of French beans, which were tinned by the pupils of the institute last autumn. Several students called attention to the unusual colour of the beans, but Fraülein Goering declared that they were quite good, and finished preparing the salad, of which she ate a large portion to show that there was nothing to fear. She was one of the first victims and died next day. Seven deaths have occurred altogether. Amongst those who were poisoned were two municipal lady nurses, Sisters Agnes and Mary, who were treated at the Municipal Hospital. A student of theology also consumed some of the dish, but he seems to have escaped. Lest he should be suddenly attacked, immediately upon hearing some of the results of partaking of the beans he took up his residence at a doctor's house so that he might have medical attention upon the first symptom.

### The Author of "Abide with Me."

AN effort is being made under the auspices of the Bishop of Exeter to complete the rebuilding of the parish church of Lower Brixham, which was begun 30 years ago in memory of the Rev. Henry Francis Lyte, author of the famous hymn "Abide with Me." A concert in aid of the movement will be given at Grosvenor House on May 10th. Mr. Lyte, who laboured among the fishermen at Brixham for a quarter of a century, refused all preferment, though he was a gifted preacher and writer. At the age of 54 he learned that he was doomed to die of consumption, and in sorrow at having to leave his work unfinished, he prayed that he might be granted to live to write something which should live to the glory of God when he was dead. His prayer was granted, for he wrote "Abide with Me" in the sunset of the last evening he ever spent at Brixham and after his last sermon in the barn-like church. A month later he died at Nice.

### Music and the Drama.

MR. GEORGE ALEXANDER, who has returned to St. James's Theatre, has revived "Old Heidelberg," the romantic comedy in which he was acting up to the time he left London last summer. This is not surprising, for there is so much that is charming in "Old Heidelberg," it is so pure and high in tone, and so admirably represented, that those who have not seen it will be glad to have the opportunity, whilst those who have already witnessed it will be glad to enjoy it again. The only alteration in the cast is that the winsome Kathie, the innkeeper's niece, instead of being played by Miss Eva Moore, is now undertaken by Miss Lilian Braithwaite, who is sweetly pathetic in the last act. Mr. Alexander as the patriotic prince, and Mr. J. D. Beveridge as the old tutor, could not give more finished performances of their respective parts.

Those who are anxious to hear the beautiful oratorio so lately written by an English composer, may be glad to know that there is to be an "Elgar Festival" at Covent Garden in March. Dr. Richter and the Hallé orchestra and chorus from Manchester will attend, and amongst the performers will be Mesdames Clara Butt, Kirkby Lunn, and Agnes Nicholls, and Messrs. Andrew Black, Ffrangçon-Davies, and Kennerley Rumford. There will be three concerts, the first consisting of "The Dream of Gerontius," the second "The Apostles," and the third orchestral, at which a new work will be produced. "The Apostles" will also be given at the Albert Hall on April 21st.

### Notes and Queries.

**FOR REGULATIONS SEE PAGE 135.**

*Travelling Expenses.*

(158) I have come all the way from London to Durham to act as nurse-companion. Should the patient pay my expenses, or I ?—*Worker.*

You should have arranged this matter before entering upon the engagement. You will now have to bear the expenses, though you might ask the patient to meet you half way.

*Truss. Prevention of Bed Sores.*

(159) *District Nurse* would be glad to know:—1. The best kind of truss for scrotal hernia in a baby. 2. The best treatment for bed-ridden case when pressure on any part produces a blister.

1. Consult a medical man. 2. See answers to examination question for nurses in The Hospital of October 31, 1903.

*Nurse-Companion.*

(160) Will you kindly tell me the best agency in London through which to obtain a post as nurse-companion ?—*Ago.*

We do not recommend private institutions. Advertise, and reply to advertisements.

Can you tell me the best way, apart from advertising, by which I could procure a post as nurse-companion ? I have put my name down at an agency, but so far I have only received two names in a month. Can you recommend an agency ?—*E. D.*

We do not recommend agencies. See reply to *Ago.*

*Abroad.*

(161) Will you kindly tell me if there is an authentic address where nurses can be put into communication with patients on the Continent or abroad, America in particular ?—*E. Le D. B.*

There are several associations which introduce nurses and patients —such as the Nurses' Co-operation, 8 New Cavendish Street, W., the Society of Chartered Nurses, 24 Prince's Street, Cavendish Square, W., etc.—but there is no special society for providing nurses with work abroad.

*Queen Alexandra's Nursing Reserve.*

(162) Will you kindly tell me to whom I should apply for particulars concerning Queen Alexandra's Nursing Reserve ?—*A. H. B.*

The address of the Army Nursing Service Reserve is 68 Victoria Street, S.W.

*Southampton.*

(163) Will you kindly tell me the names of the two largest general hospitals in Southampton ?—*V. L.*

The only general hospital at Southampton is the Royal South Hants and Southampton Hospital.

*Children's Nurse.*

(164) 1. Will you kindly tell me where ladies are trained as children's nurses ? 2. What length of time is required ? 3. Is a hospital training necessary ?—*M. E. D.*

1. The Norland Institute, 10 Pembridge Square, W.; and the Sesame House for Home Life Training, 43 Acacia Road, St. John's Wood ; The Home for Children, Court Royal, South Norwood Hill, S.E. 2. Write for particulars and terms. 3. No hospital training is required.

*Hospital Training.*

(165) Will you kindly tell me if three years' training in a workhouse infirmary renders a nurse eligible for an appointment at one of the large general hospitals of London, or for important posts in other branches of the nursing profession. Is she equal to a nurse trained in a general hospital ?—*A. B. R.*

The training at the best workhouse infirmaries is recognised by the large general hospitals in London.

**Important Nursing Textbooks.**

**"The Nursing Profession: How and where to Train." 2s. net; 2s. 4d. post free.**

**"A Handbook for Nurses." By Dr. J. K. Watson. 5s. net; 5s. 4d. post free.**

**"Practical Guide to Surgical Bandaging and Dressings." By Wm. Johnson Smith, F.R.C.S. 2s. post free.**

**"The Nurses' Dictionary of Medical Terms and Nursing Treatment." By Honnor Morten. 2s. post free.**

**"The Human Body: its Personal Hygiene and Practical Physiology." By B. P. Colton. 5s. post free.**

**"Art of Feeding the Invalid." (Popular Edition). 1s. 6d. post free.**

**"On Preparation for Operation in Private Houses. By Stanhope Bishop, F.R.C.S. 6d. post free**

### For Reading to the Sick.

"ABIDE IN ME."

If thou unto the Lord wilt upright be,
No cloud or darkness can with thee abide,
For oft in such He loves His face to hide;
'Tis His pavilion, which no eye can see;
His secret presence where He covereth thee
To keep thee safe from harm on every side;
A sure defence the righteous oft have tried
Who know His loving words "Abide in Me."
Abide in Thee! O Lord in me abide;
Renew in me each day Thy heavenly grace;
Make me submissive to Thy holy Will,
And in Thy power learn ever to confide.
So shall my soul, when storms may hide Thy face
Still trust in Thee who sayest, "Peace, be still."

*C. Elcock.*

Yea! In Thy life our little lives are ended,
Into Thy depths our trembling spirits fall;
In Thee enfolded, gathered, comprehended,
As holds the sea her waves—Thou hold'st us all.

*E. Scudder.*

If thou wishest to live with and to God, and that God should dwell more in thee and be thy God, these few brief rules may help thee.

Be with God in thy outward works; refer them to Him, offer them to Him, seek to do them in Him and for Him, and He will be with thee in them, and they shall not hinder, but rather invite His Presence into thy soul.—*E. B. Pusey.*

Watch the sun drawing up the mists into its own brightness; watch the river as it flows, absorbing all lesser streams; watch the flame as it stretches forth and kindles the fuel it meets with. More than all these does God absorb into Himself, into an ineffable, unspeakably sweet union, the soul which really loves Him. It is a union, not bodily, but spiritual; a union to be obtained by seeking God with the understanding and with the will. St. Bernard says that there are four principal means of attaining this union of the mental faculties with God—spiritual reading, meditation, prayer, and contemplation. These he calls the steps by which the devout soul rises to God; and he adds, reading walks, meditation runs, prayer flies, and contemplation attains the end of the whole, and rests in God Himself.

*S. Lear.*

Because all those scattered rays of beauty and loveliness which we behold spread up and down over all the world, are only the emanations of that inexhausted light which is above; therefore should we love them all in that, and climb up always by those sunbeams unto the eternal Father of lights: we should look upon Him, and take from Him the pattern of our lives, and always eying Him, should, as Hierocles speaks, "polish and shape our souls into the clearest resemblance of Him."—*Dr. John Smith,* 1652.

Just to follow hour by hour
As He leadeth;
Just to draw the moment's power
As it needeth.

*F. R. Havergal.*

